# Personalised cancer follow-up: risk stratification, needs assessment or both?

**DOI:** 10.1038/bjc.2012.108

**Published:** 2012-04-03

**Authors:** T Filleron, F Dalenc, A Kramar

**Affiliations:** 1 Institut Claudius Regaud, 20–24, rue du Pont Saint Pierre, F-31052 Toulouse, France; 2 Centre Oscar Lambret, 3 rue Frédéric Combemale, 59020 Lille, France

**Sir**,

We read with great interest the editorial by Watson *et al* (2012) on the importance of proposing personalised follow-up for cancer survivors. Usage of this terminology in itself implies cure of cancer. However, the editorial is focused on stratifying patients according to the probability of a harmful effect, especially medical and psychosocial. Current risk stratification focuses primarily on the risk of recurrence as identified in a 2007 survey addressed to patients and professionals in hospital and primary care. Other reasons include the management of early complications and late effects of treatment (the National Cancer Survivorship Initiative, Rapid Review of Follow-Up).

Post therapeutic follow-up, however, has many objectives, which are all just as important as detecting curable disease relapses or side effects: (1) social aspects having to do with professional reinsertion and rehabilitation are a regular concern for many cancer survivors. Hewitt *et al* (2007); (2) economic aspects having to do with the number and frequency of visits concerning the optimal use of resources (Leiter *et al*, 2009); and (3) scientific aspects having to do with evaluating therapeutic results of treated patients in observational studies.

Proposing personalised follow-up visits by taking account of all these aspects is just as important as is the decision of prescribing individualised treatment. Some guidelines recommend planning follow-up visits only in the case of symptom manifestation. In any case, results from an audit of practices conducted by the REACT group of the ESTRO concerning the usefulness of current follow-up practice after radiotherapy treatment pointed out the fact that patients attributed symptoms more often to their disease whereas the physician attributed them more often to treatment (Ataman *et al*, 2006). This paper recommends to propose adapted education to the patients on the risk of side effects, and to concentrate follow-up assessments on patients’ support. In order to stratify follow-up according to the risk of side effects, it is necessary to identify which patients are at risk and at what time point they are most likely to exhibit symptoms. As well, for the optimal identification of patients at risk, there is a need to better understand mechanisms that lead to chronic toxicities. In order to provide a possible solution to this problem it is essential to analyse large databases from clinical trials with homogeneous treatment modalities using adequate statistical methodology. Nevertheless, one of the limitations of institutional databases is that although the acute side effects of cancer treatment are well described, the chronic harmful effects, including alterations of quality of life, are not well investigated. It is also possible to design specific trials to study prognostic factors associated with specific side effects, such as the FATSEIN study that aims to identify the determinants of cancer-related fatigue (Rotonda *et al*, 2011). Moreover, it may be necessary to take into account not only the occurrence of a particular effect, but also its timing, its severity and its possible recurrent nature. For evaluating long-term treatment effects, prevalence is just as important as incidence, as this function takes account of reversible and transient events. It is defined as the proportion of patients occupying a particular state of health at a particular time point (Pepe *et al*, 1991; Bentzen *et al*, 2003). Using this methodology, it should be possible to propose more individualised follow-up for future patients according to the incidence and prevalence of the events of interest.

Concerning the stratification of patients according to prognosis, we agree that this question is largely open to debate in the medical community. Actual surveillance calendars do not always take into account the dynamics of event times. Also, prognostic factors are not usually used to identify patients who are more or less at risk of relapse over time. Certain patients will never relapse during their entire surveillance period, whereas others may present relapses rather early after the end of their initial treatment. Different strategies of follow-up should be used, not only with intensive or less intensive exams but also a better spacing out of visits. At the present time, most follow-up visits are planned in a uniform manner, either annually or semi-annually depending on the type of cancer, even though, for some cancer sites >50% of patients will never relapse no present any side effects. Several statistical methods are available to adapt follow-up to individual patient profiles. We recently developed a two-stage strategy by first identifying prognostic factors associated with time to failure (recurrence, complications, and so on), and then proposing a scheduling of visits using quantile estimates of the cumulative incidence or cumulative risk of relapse (Filleron *et al*, 2009). This method permits a better spacing of visits in planning follow-up visits around the time points where events are most likely to occur. However, this method depends on the type of events to be detected and does not define a total length of follow-up, but which could be implemented according to methods developed by Mould *et al* (2004). They present an example where they use a simple formula to predict how long oncologist should follow patients with early stage breast cancer after treatment.

It thus seems that personalised cancer follow-up is just as important as personalised treatment. There is no specific reason to propose frequent visits just after treatment for patients who have responded well to treatment. In fact the majority of patients presenting early relapse are more likely to have had advanced disease for which there is no potential curative treatment. On the other hand, it is just as unnecessary to plan annual follow-up visits with imaging tools for good risk testicular cancer patients for 20 years, for example, as very few patients are expected to have relapsed after 2 years. The economic resources of health care are limited and the incidence and prevalence of cancer is increasing. So, it has become a public health problem to propose adapted follow-up strategies in order to optimise both economic and physician resources.
